# Prognostic value of long-term antidiabetic and antihypertensive therapy in postoperative gastric cancer patients: the FIESTA study

**DOI:** 10.1186/s12876-022-02514-4

**Published:** 2022-10-09

**Authors:** Laicheng Wang, Dan Hu, Zongcheng Fan, Jianjian Yu, Shunpeng Zhang, Yunchai Lin, Xin Chen, Xiandong Lin, Xiyao Yan, Jinxiu Lin, Feng Peng

**Affiliations:** 1 Department of Cardiology, The First Affiliated Hospital of Fujian Medical University, Chazhong Road 20, Fuzhou, 350005 Fujian China; 2 Department of Pathology, Clinical Oncology School of Fujian Medical University, Fujian Cancer Hospital, Fuma Road 420, Fuzhou, 350000 Fujian China; 3 Laboratory of Radiation Oncology and Radiobiology, Clinical Oncology School of Fujian Medical University, Fujian Cancer Hospital, Fuma Road 420, Fuzhou, 350000 Fujian China

**Keywords:** Gastric cancer, Hypertension, Diabetes mellitus, Prognosis, Mortality, FIESTA study

## Abstract

**Background:**

Gastric cancer is often comorbid with hypertension and diabetes mellitus and increases the mortality risk.

**Materials and methods:**

We conducted this prospective cohort study to investigate antidiabetics and antihypertensives’ impact on gastric cancer survival. 3012 patients with gastric carcinoma undergoing radical gastrectomy were enrolled since January 2000 and followed up until July 2020.

**Results:**

Hypertension and diabetes patients had worse survival than patients without hypertension and diabetes [median survival time (MST): 48 versus 112.5 months, *p* < 0.001 for hypertension, MST: 32.7 versus 183+ months, *p* < 0.001 for diabetes]. Compared to untreated patients, treated patients had better survival (MST: 109.7 months versus 39.1 months, *p* < 0.001 for antihypertensives, MST: 120.9 months versus 22.3 months, *p* < 0.001 for antidiabetics). Antihypertensives and antidiabetics were related to 42% (HR 0.58, 95% CI 0.47–0.73, *p* < 0.001) and 70% (HR 0.30, 95% CI 0.24–0.38, *p* < 0.001) reduced mortality risk relative to those without medications. metformin and Calcium channel blockers can better-improved prognosis compared to others (*p* = 0.00029 and *p* = 0.015).

**Conclusion:**

Post-surgical gastric cancer patients could benefit substantially from anti-diabetes and antihypertensive therapy. Metformin and Calcium channel blockers may be superior to other medications.

**Supplementary Information:**

The online version contains supplementary material available at 10.1186/s12876-022-02514-4.

## Introduction

Gastric carcinoma (GC) is the fifth most prevalent cancer and the third leading cause of cancer death worldwide [1, 2]. Particularly in China, which accounts for nearly half of the global incidence and cancer-related deaths of gastric carcinoma, the prognosis of gastric cancer remains unfavorable [2, 3]. Consequently, finding rational and practical strategies to reduce mortality risk and prolong survival is strongly required.

Hypertension (HT) and diabetes mellitus (DM) are the most common comorbidities of GC [4, 5]. Moreover, the impact of HT and DM on cancer prognosis has been extensively studied [6]. Meta-analysis revealed that DM doubles the mortality risk of GC patients, and HT significantly increases overall mortality and cancer-related mortality [7, 8]. Likewise, our previous reports suggested that hyperglycemia and elevated blood pressure were significant predictive figures for mortality of GC patients [9].

Nevertheless, the impact of antihypertensive and antidiabetic medication on GC prognosis remains controversial. Cohort studies suggested that metformin was related to a lower recurrence and higher survival rate of GC [10, 11]. Parallelly, a recent study suggested that verapamil improved the overall survival of late-stage gastric patients under chemotherapy [12]. Conversely, several cohort studies found no extra benefit from antidiabetic therapy on cancer survival [13, 14].

We explored the long-term antihypertensive and antidiabetic therapy efficacy on GC patients’ prognosis through the detabase of the Fujian prospective investigation of cancer (FIESTA) study.

## Materials and methods

### The FIESTA study

The FIESTA study is a long-term prospective study to assess risk factors for death in patients with common gastrointestinal cancers (including esophagus, stomach, and colon) undergoing surgery [9, 15–17].

### Inclusion and exclusion criteria

Since January 2000, patients with gastric carcinoma who underwent radical gastrectomy were recruited from the Department of Thoracic Surgery, Fujian Provincial Cancer Hospital. The most recent follow-up was in July 2020. Non-Han Chinese population and patients with previous radical gastrectomy, preoperative chemotherapy, or radiotherapy were excluded. Only participants who were followed up for more than one month and with complete data on HT or DM were analyzed.

### Patient characteristics and diagnosis

Participants completed a self-designed questionnaire containing age, gender, tobacco and alcohol histories, and family history of tumors. Certified examiners measured blood pressure by mercury sphygmomanometers three times every 5 min. After fasting for 8–12 h, patients’ venous blood samples were collected before surgery to examine blood routine tests, blood glucose, blood lipids, and other biochemical indicators according to a standard procedure. During surgery, participants’ cancerous and normal gastric tissue samples were taken to evaluate the pathological characteristics of the tissue. Antihypertensive and antidiabetic medication use were according to self-report during the follow-up. Antihypertensives were classified as calcium channel blockers (CCB), angiotensin-converting enzyme inhibitors (ACEI), angiotensin receptor blocker (ARB), diuretics, beta-receptor antagonists (β-blocker), and unclear. Antidiabetics were classified as metformin, sulfonylurea, glucosidase inhibitor, insulin, and unclear.

Diagnose of HT and DM were based on physical examination and venous blood test preoperative and during the follow-up. HT was defined as systolic blood pressure ≥ 140 mmHg and/or diastolic blood pressure ≥ 90 mmHg, antihypertensive medication use, or former medication records. DM was defined as fasting plasma glucose ≥ 7.0 mmol/L, positive OGTT test (2 h glucose ≥ 11.1 mmol/L), antidiabetic medication use, or former medication records. GC was diagnosed through pathological examination.

### Follow-up and outcome assessments

The annually follow-up assessment of GC patients, mainly through outpatient check-ups, telephone, or postal mails. We acquired death dates through family members or medical reports. The primary outcome was overall mortality. Survival time was calculated from the date of receiving radical resection until death or last follow-up.

### Statistical analysis

Baseline characteristics were compared by chi-square test for Categorical/dichotomic variables and ANOVA or Kruskal–Wallis test for continuous variables. Kaplan–Meier curves and Log-rank tests were used to present and test the differences in cumulative survival rates. The effect of antihypertensive and antidiabetic medication on mortality risk was applied by Cox proportional hazards regression. Risk prediction assessment was expressed by hazard ratio (HR) and 95% confidence interval (95% CI). All statistical analyses were done by the STATA 16.0 (StataCorp, College Station, TX, USA) and R 4.1.1.

## Results

### Baseline characteristics

A total of 3012 participants have completed the follow-up ranging from 1.1 months to 183.3 months (median: 44.05 months). Baseline characteristics are summarized in Tables 1 and 2 (additional comparisons between baseline characteristics are in Additional file 1: Table S1 and Table S2). Among them, 312 and 497 patients had treated and untreated hypertension. Meanwhile, 243 and 733 patients had treated and untreated diabetes. Patients without HT and DM were significantly younger and with lower blood pressure, fasting plasma glucose (FPG), blood lipids, and body mass index (BMI) than hypertensive and diabetic patients. Plus, untreated HT patients have higher systolic blood pressure, and untreated DM patients have significantly higher systolic blood pressure and FPG than treated patients. Besides that, the untreated DM group had more stage IV and underwent chemotherapy patients than the other two groups. No other characteristic significance was found across the three groups.

**Table 1:** Baseline characteristics of gastric cancer patients per hypertension and medications

Characteristics	no HT	Treated HT	Untreated HT	*P*
Number	2203	312	497	
Age (year)	56.92 (11.31)	64.51 (9.2)	61.50 (10.46)	< 0.001
Male (N%)	1636 (74.26%)	230 (73.71%)	373 (75.05%)	0.904
Smoking (N %)	409 (20.70%)	63 (20.25%)	84 (16.90%)	0.158
Alcohol (N %)	118 (5.98%)	20 (6.43%)	30 (6.03%)	0.955
Family history (N %)	178 (9.03%)	36 (11.61%)	44 (8.85%)	0.324
BMI (kg/m^2^)	22.44 (2.98)	24.33 (3.13)	23.35 (3.03)	< 0.001
CCB (N%)	NA	149 (50.96%)	NA	
ACEI/ARB (N%)	NA	52 (16.67%)	NA	
Diuretic (N%)	NA	7 (2.24%)	NA	
b-blocker (N%)	NA	15 (4.81%)	NA	
Unclear (N%)	NA	116 (37.18%)	NA	
SBP (mmHg)	115.11 (12.06)	144.3 (13.63)	152.35 (14.46)	< 0.001
DBP (mmHg)	72.55 (8.63)	88.16 (9.04)	88.58 (10.43)	< 0.001
FPG (mmol/L)	5.93 (2.27)	6.9 (2.63)	6.83 (2.86)	< 0.001
TG (mmol/L)	1.17 (0.87)	1.32 (0.85)	1.31 (0.95)	< 0.001
TC (mmol/L)	4.46 (1.04)	4.69 (1.1)	4.67 (1.14)	< 0.001
HDL (mmol/L)	1.05 (0.40)	1.02 (0.37)	1.01 (0.37)	0.011
LDL (mmol/L)	2.92 (0.90)	3.16 (0.9)	3.11 (0.97)	< 0.001
TNM stage (N%)				0.052
I	248 (12.18%)	39 (12.50%)	57 (11.52%)	
II	308 (15.13%)	53 (16.99%)	63 (12.73%)	
III	1167 (57.32%)	178 (57.05%)	271 (51.75%)	
IV	313 (15.37%)	42 (13.46%)	104 (21.01%)	
Pathological type (N%)	0.696			
adenocarcinoma	1519 (76.87%)	243 (78.90%)	385 (78.09%)	
signet-ring cell	424 (21.46%)	58 (18.83%)	100 (20.28%)	
neuroendocrine	16 (0.81%)	3 (0.97%)	6 (1.22%)	
Other	17 (0.86%)	4 (1.30%)	2 (0.41%)	
Differentiated				0.682
High	22 (1.11%)	6 (1.95%)	6 (1.22%)	
Middle	745 (37.72%)	121 (39.29%)	180 (36.51%)	
Low	1208 (61.16%)	181 (58.77%)	307 (62.27%)	
Chemotherapy (N %)	700 (31.77%)	101 (32.4%)	151 (30.4%)	0.792
Chemotherapy courses (N)	3.67 (2.4)	3.32 (2.3)	3.40 (2.3)	0.161

Data are represented as mean (standard deviation) or count (percentage%)

Chi-square test for Categorical variables and ANOVA or Kruskal–Wallis test when nonparametric for continuous variables were used

HT, hypertension; DM, diabetes mellitus; Alcohol: alcohol use history; Smoking: tobacco use history; Family history, Family history of cancer; BMI, Body mass index; CCB: calcium channel blockers, ACEI: angiotensin-converting enzyme inhibitors, ARB: angiotensin receptor blocker, β-blocker: beta-receptor antagonists; SBP, systolic blood pressure; DBP, diastolic blood pressure; FPG, fasting plasma glucose; TG, Triglyceride; TC, Total Cholesterol; LDL, Low-density Lipoprotein; HDL, High-density Lipoprotein

**Table 2:** Baseline characteristics of gastric cancer patients per diabetes mellitus and medications

Characteristics	no DM	Treated DM (	Untreated DM	*P*
Number	2036	243	733	
Age (year)	57.95 (11.26)	61.43 (10)	59.19 (11.54)	< 0.001
Male (N%)	1551 (76.18%)	179 (73.66%)	509 (69.44%)	0.002
Smoking (N %)	381 (21.12%)	44 (18.11%)	131 (17.90%)	0.137
Alcohol (N %)	120 (6.66%)	19 (7.82%)	29 (3.96%)	0.017
Family history (N %)	178 (9.87%)	26 (10.70%)	54 (7.39%)	0.109
BMI (kg/m2)	22.43 (2.86)	24.02 (3.4)	23.35 (3.28)	< 0.001
Metformin (N %)	NA	170 (69.96%)	NA	
Sulfonylurea (N %)	NA	54 (22.22%)	NA	
Glucosidase inhibitor (N %)	NA	4 (1.65%)	NA	
Insulin (N %)	NA	5 (2.06%)	NA	
Unclear (N %)	NA	22 (9.05%)	NA	
SBP (mmHg)	122.46 (18.65)	126.49 (19.61)	133.65 (21.77)	< 0.001
DBP (mmHg)	76.51 (11.16)	77.92 (11.47)	78.42 (12.94)	0.001
FPG (mmol/L)	4.87 (0.74)	8.49 (2.61)	9.15 (2.67)	< 0.001
TG (mmol/L)	1.09 (0.71)	1.55 (1.27)	1.37 (1.05)	< 0.001
TC (mmol/L)	4.49 (1)	4.59 (1.03)	4.57 (1.24)	< 0.001
HDL (mmol/L)	1.1 (0.35)	0.92 (0.34)	0.93 (0.47)	0.011
LDL (mmol/L)	2.95 (0.88)	2.99 (0.92)	3.04 (1.02)	0.293
TNM stage (N%)				< 0.001
I	280 (14.97%)	30 (12.35%)	34 (4.66%)	
II	314 (16.78%)	38 (15.64%)	72 (9.88%)	
III	1048 (56.01%)	146 (60.08%)	422 (57.89%)	
IV	229 (12.24%)	29 (11.93%)	201 (27.57%)	
Pathological type (N%)				0.252
Adenocarcinoma	1426 (78.74%)	181 (74.79%)	540 (74.59%)	
Signet-ring cell	357 (19.71%)	54 (22.31%)	171 (23.62%)	
Neuroendocrine	14 (0.77%)	4 (1.65%)	7 (0.97%)	
Other	14 (0.77%)	3 (1.24%)	6 (0.83%)	
Differentiated				< 0.001
High	25 (1.38%)	3 (1.24%)	6 (0.83%)	
Middle	734 (40.55%)	90 (37.19%)	222 (30.66%)	
Low	1051 (58.07%)	149 (61.57%)	496 (68.51%)	
Chemotherapy (N %)	608 (29.8%)	101 (32.4%)	151 (30.4%)	0.010
Chemotherapy courses (N)	3.51 (2.4)	4.09 (2.4)	3.61 (2.3)	0.078

Data are represented as mean (standard deviation) or count (percentage%)

Chi-square test for Categorical variables and ANOVA or Kruskal–Wallis test when nonparametric for continuous variables were used. HT, hypertension; DM, diabetes mellitus; Alcohol: alcohol use history; Smoking: tobacco use history; Family history, Family history of cancer; BMI, Body mass index; CCB: calcium channel blockers, ACEI: angiotensin-converting enzyme inhibitors, ARB: angiotensin receptor blocker, β-blocker: beta-receptor antagonists; SBP, systolic blood pressure; DBP, diastolic blood pressure; FPG, fasting plasma glucose; TG, Triglyceride; TC, Total Cholesterol; LDL, Low-density Lipoprotein; HDL, High-density Lipoprotein

### Survival comparison

**Fig. 1:**
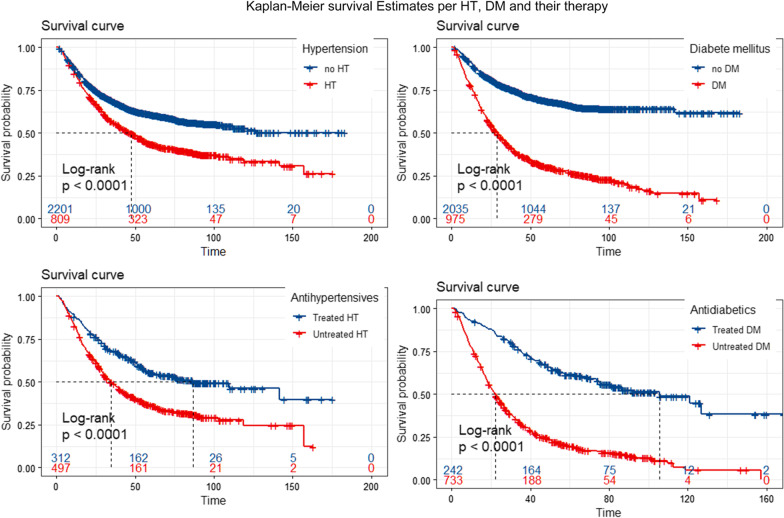
Kaplan–Meier survival curves per hypertension (the upper left panel) and diabetes mellitus (the lower left panel), antihypertension medication (the upper right panel), and antidiabetic medication (the lower right panel). HT, hypertension; DM, diabetes mellitus;

The comparison of cumulative overall survival rates across groups was provided in Fig. 1. Not unexpectedly, patients with DM and HT had remarkably poorer survival duration than non-hypertension and non-diabetic patients (MST: 48 versus 112.5 months, Log-rank test *p* < 0.001 for HT, MST: 32.7 versus 183+ months, *p* < 0.001 for DM).

Moreover, antihypertensive and antidiabetic therapies were significantly associated with improved prognosis and prolonged survival time (MST: 109.7 versus 39.1 months, *p* < 0.001 for antihypertensive, MST: 120.9 versus 22.3 months, *p* < 0.001 for antidiabetics). The combined effect of hypoglycemic and antihypertensive on gastric cancer survival is presented in Additional file 1: Fig. S1.

### Mortality risk estimation

To further explore the effect size of hypoglycemic and antihypertensive therapy on GC patients’ prognosis. After the proportional-hazards assumption was satisfied, overall and stratified Cox regression was conducted to derive survival estimates (Additional file 1: Fig. S2).

**Fig. 2:**
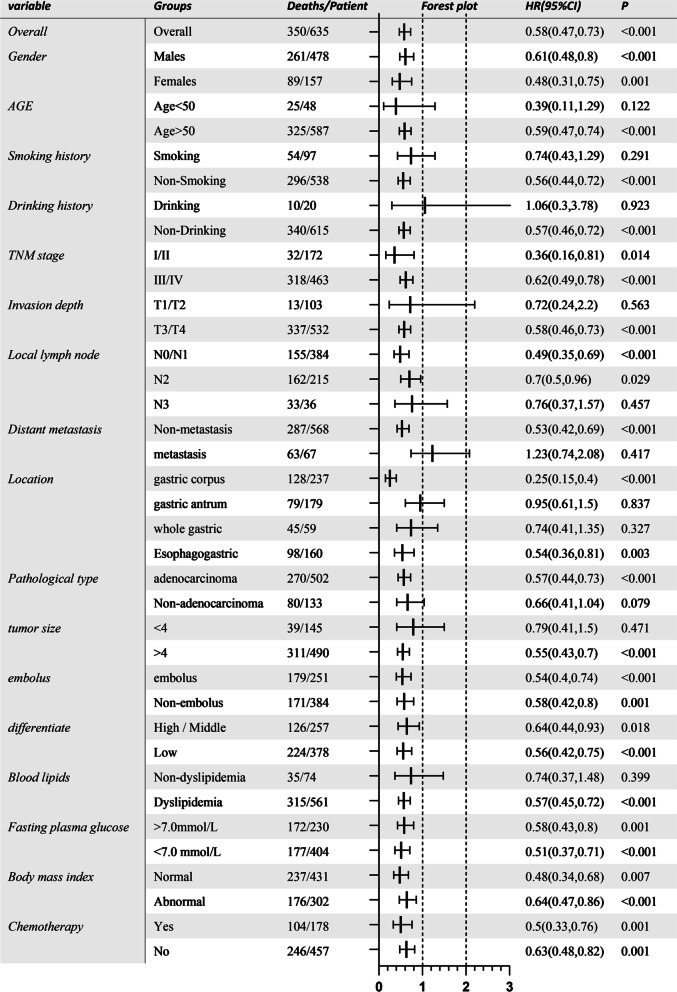
Overall and stratified analyses of effect with antihypertension therapy on mortality risk of post-surgical gastric cancer

**Fig. 3:**
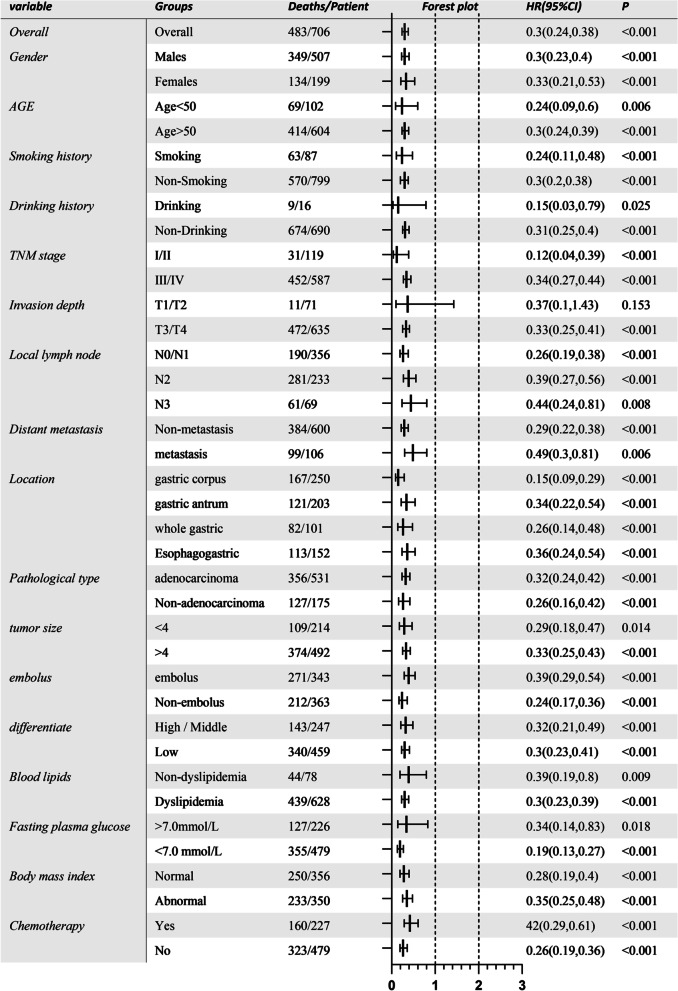
Overall and stratified analyses of effect with antidiabetic therapy on mortality risk of post-surgical gastric cancer

As presented in Figs. 2, 3, and Additional file 1: Table S3, after adjusting for age, gender, tobacco and alcohol use history, and family history of tumor, the overall mortality risk of patients with antihypertensive and hypoglycemic therapies were lower by 42% (HR 0.58, 95% CI 0.47–0.73, *p* < 0.001) and 70% (HR 0.30, 95% CI 0.24–0.38, *p* < 0.001).

In stratified analysis by personal and family history, only patients without drinking and smoking history and age more than 50 years reduce significant risk in the antihypertensive group. By blood routine test and biochemical indicators, only patients with dyslipidemia, and blood type A/O, white blood cell counts between 4 and 10*10^9/L, and platelet under 300*10^9/L reduced significant mortality risks. All stratified groups with antidiabetic reduced significant risks despite stratification of blood routine tests and biochemical indicators, personal and family history. Noteworthily, gastric cancer patients with distant metastasis, tumor size under 4 cm, non-adenocarcinoma type, sited in the gastric antrum and whole gastric did not receive significant advantage undertaken antihypertensives. By contrast, there was significant risk reduction for antidiabetic therapy in all patients despite TNM stages, tumor sites, pathological types, and tumor size, except for patients with T1/T2 stages.

### Survival comparison of medications

**Fig. 4:**
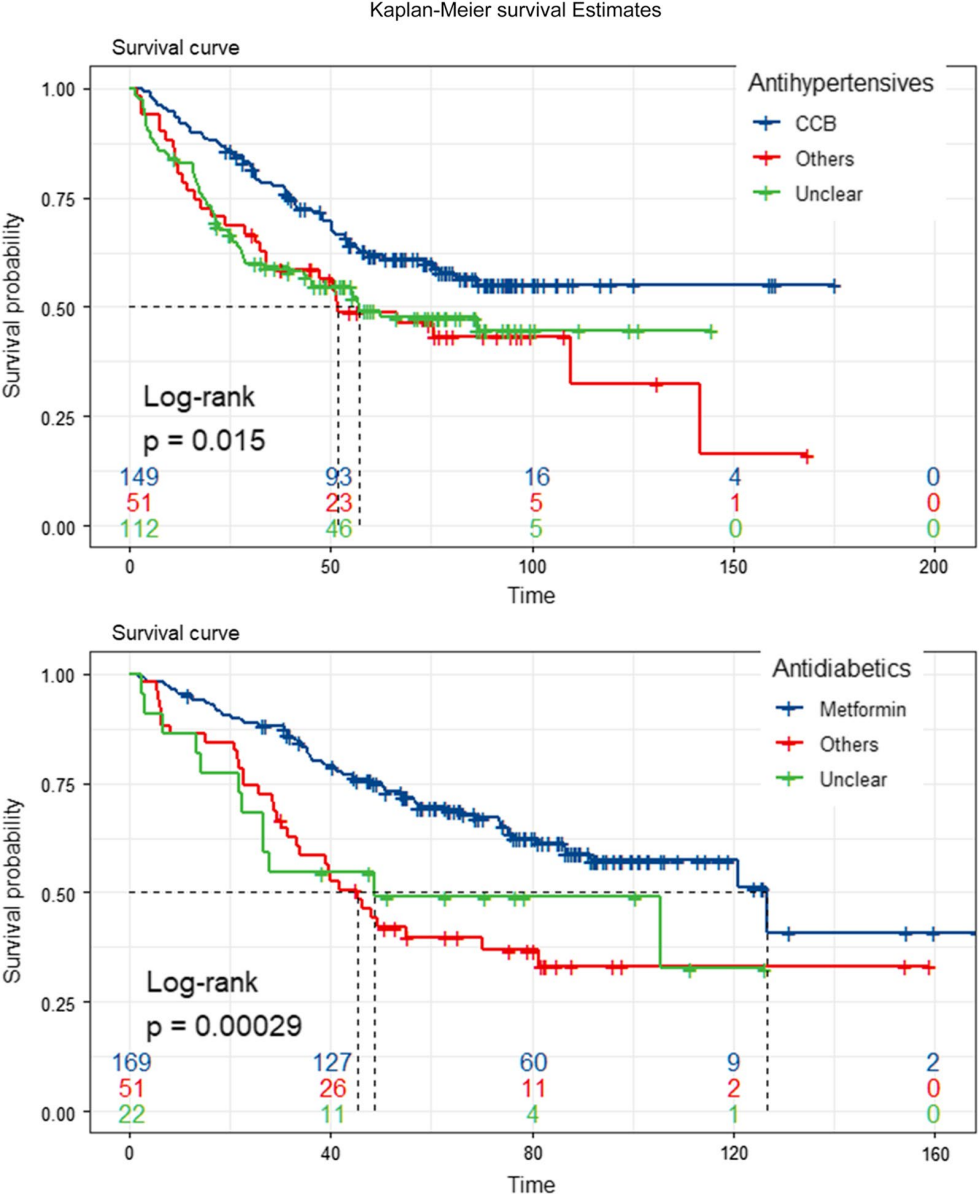
Kaplan–Meier survival curves per antihypertension medication (the upper panel) and antidiabetic mellitus medication. CCB, calcium channel blockers; Others, patients undertaken other medication; Unclear, not knowledge of medication type

Figure 4 and Additional file 1: Table S4 represent the efficacy of each antidiabetic and antihypertensive medication on cumulative survival rates. After being stratified by antihypertensives, CCB users had a significantly better survival rate after surgery than those who received other medications: the HR for CCB is 0.33, the HR for other antihypertensives was 0.71 and the log-rank test among groups was 0.015. Similarly, after stratification by antidiabetic medications, gastric cancer patients who received metformin had a better survival rate than patients who received other antidiabetics: the HR for metformin is 0.16, the HR for other antidiabetics was 0.78 and the log-rank test among groups were 0.00029. Moreover, in co-existing DM and HT patients, CCB and metformin better reduced mortality risk compared to other medications (HR = 0.41and 0.18, respectively).

## Discussion

In this present study, we found that co-existing DM or HT are related to significant mortality risk in post-surgical gastric cancer patients. Whereas antidiabetic and antihypertensive therapy can remarkably improve GC patient outcomes. Our finding supports the needing for enhanced screening and targeted intervention of HT and DM in all GC patients.

Numerous epidemiological evidences support that DM and HT could increase cancer mortality [6, 8, 18]. In supporting our findings, a meta-analysis including 66 studies indicated that HT was associated with a 20% overall mortality risk and a 12% cancer-specific mortality risk increased [7]; our findings suggested that antihypertensive intervention can prolong survival time and reduce overall mortality risk by 42% of GC patients. Meta-analysis demonstrated that DM could significantly increase GC risk and mortality [19]; our results further revealed that antidiabetic intervention could reduce the 70% mortality risk of GC patients. Antihypertensive and antidiabetic medication may improve GC prognosis by several means. First of all, decreased glucose suppresses tumor growth and metastasis [20, 21]. Additionally, hypertension and hyperglycemia correlated to more anti-cancer cardiovascular complications [22, 23]. Furthermore, studies have suggested that antidiabetics and antihypertensives may increase the efficacy of chemotherapy [24].

Another important finding is that metformin and CCB can better-improved prognosis compared to other medications. A recent study revealed that metformin could activate adenosine 5’-monophosphate-activated protein kinase (AMPK) by inhibiting the lysosomal proton pump vacuolar-type ATPase [25], and the AMPK pathway has been found to promote apoptosis in tumor cells [26]. Metformin can regulate the expression of programmed cell death 1 ligand 1 (PD-L1) to enhance the immune response against cancer [27]. Some scholars support that metformin can reduce the helicobacter pylori infection to inhibit gastric cancer invasion and migration [28]. Therefore, metformin users may obtain more benefits other than blood glucose reduction. Merely a few studies have focused on the antitumor effect of CCB. Though, recent studies have indicated that CCB could specifically repress voltage-gated Ca+ channels (VGCCs) and other pathways to suppress gastric cancer cell growth [29]. Besides that, observation studies demonstrated that CCB is also associated with lower risk and better prognosis of cancer [12, 30]. Nevertheless, Further investigation is awaited.

Several limitations of our study need to be considered. Foremost, this study spans more than ten years; in the meantime, profoundly surgical techniques in advance might introduce a possible bias, which may underestimate the influence of antihypertensives or antidiabetics. Secondly, patients with untreated diabetes tend to have more progressed tumors, which may overestimate the impact of antidiabetics. Finally, this study merely included post-operation patients from a single-center, which made the finding lack external authenticity among other gastric patients.

## Conclusion

After all, post-surgical gastric cancer patients could benefit substantially from anti-diabetes and antihypertensive therapy. Metformin and Calcium channel blockers may be superior to other medications in gastric cancer patients. This study emphasized that supporting screening programs and pharmacological treatment for diabetes mellitus and hypertension is critical to prolonging the survivorship of gastric cancer patients.

## Supplementary Information

**Additional file 1**. Supplementary materials.

## Data Availability

The datasets generated during and/or analysed during the current study are available from the corresponding author on reasonable request.
