# Supplementary material for: Prognostic value of long-term antidiabetic and antihypertensive therapy in postoperative gastric cancer patients: the FIESTA study
Source: BMC Gastroenterol. 2022 Oct 9;22:429. doi: 10.1186/s12876-022-02514-4 (PMC9549639; doi:10.1186/s12876-022-02514-4)
Supplement: Supplementary file 1 — Additional file 1. Supplementary materials. [file 12876_2022_2514_MOESM1_ESM.docx]

| TableS1: Additional baseline characteristics of gastric cancer patients per hypertension | | | | |
| --- | --- | --- | --- | --- |
| Characteristics | no HT | Treated HT | Untreated HT | P |
| Number | 2203 | 312 | 497 |  |
| Invasion depth (N%) | | | | 0.765 |
| T1 | 183 (89.50%) | 27 (8.65%) | 44 (8.87%) |  |
| T2 | 182 (8.93%) | 32 (10.26%) | 41 (8.27%) |  |
| T3 | 1112 (54.54%) | 180 (57.69%) | 280 (56.45%) |  |
| T4 | 562 (27.56%) | 73 (23.40%) | 131 (26.41%) |  |
| Lymph node metastasis (N%) | | | | 0.23 |
| N0 | 552 (27.07%) | 88 (28.21%) | 119 (23.99%) |  |
| N1 | 631 (30.95%) | 110 (35.26%) | 155 (31.25%) |  |
| N2 | 667 (32.71%) | 90 (28.85%) | 182 (36.69%) |  |
| N3 | 189 (9.27%) | 24 (7.69%) | 41 (8.27%) |  |
| Distant metastasis (N%) | 251 (11.39%) | 32 (10.26%) | 73 (14.69%) | 0.081 |
| Pathological type (N%) | | | | 0.696 |
| adenocarcinoma | 1519 (76.87%) | 243 (78.90%) | 385 (78.09%) |  |
| signet-ring cell | 424 (21.46%) | 58 (18.83%) | 100 (20.28%) |  |
| neuroendocrine | 16 (0.81%) | 3 (0.97%) | 6 (1.22%) |  |
| Other | 17 (0.86%) | 4 (1.30%) | 2 (0.41%) |  |
| Location (N%) | | | | <0.001 |
| gastric corpus | 724 (36.64%) | 91 (29.64%) | 216 (43.81%) |  |
| gastric antrum | 601 (30.41%) | 80 (26.06%) | 142 (28.80%) |  |
| whole gastric | 225 (11.39%) | 42 (13.68%) | 43 (8.72%) |  |
| esophagogastric junction cancer | 426 (21.56%) | 94 (30.62%) | 92 (18.66%) |  |
| blood type (N%) | | | | 0.919 |
| O | 783 (39.75%) | 130 (41.94%) | 203 (40.13%) |  |
| A | 585 (29.62%) | 95 (30.65%) | 144 (29.63%) |  |
| B | 463 (23.44%) | 62 (20.00%) | 113 (22.94%) |  |
| AB | 144 (7.29%) | 23 (7.42%) | 36 (7.30%) | ANOVA |
| WBC (10^9/L) | 6.65 (2.76) | 6.8 (2.02) | 6.68 (3.65) | 0.6784 |
| N (10^9/L) | 4.03 (2.59) | 4.21 (0.018) | 4.03 (0.0188) | 0.4673 |
| Ly (10^9/L) | 1.91 (1.19) | 1.91 (0.88) | 1.78 (0.64) | 0.0535 |
| RBC (10^12/L) | 4.5 (9.71) | 5.5 (22.5) | 4.4 (5.11) | 0.329 |
| PLT (10^9/L) | 267.61 (98.88) | 264.32 (92.96) | 258.61 (88.89) | 0.1759 |

Chi-square test and ANOVA or Kruskal-Wallis test when nonparametric were used

Abbreviations: WBC, White blood cell count; N Neutrophil count; Ly, Lymphocyte count; RBC, Red blood cell count; PLT, Platelet count;

| TableS2: Additional baseline characteristics of gastric cancer patients per diabetes mellitus and medication | | | | |
| --- | --- | --- | --- | --- |
| Characteristics | no DM | Treated DM | Untreated DM | P |
| Number | 2036 | 243 | 733 |  |
| Invasion depth (N%) | | | | <0.001 |
| T1 | 205 (10.95%) | 22 (9.05%) | 27 (3.69%) |  |
| T2 | 193 (10.31%) | 24 (9.88%) | 38 (5.19%) |  |
| T3 | 1029 (54.97%) | 132 (54.32%) | 411 (56.15%) |  |
| T4 | 445 (23.77%) | 65 (26.75%) | 256 (34.97%) |  |
| Lymph node metastasis (N%) | | | | <0.001 |
| N0 | 595 (31.77%) | 63 (25.93%) | 101 (13.80%) |  |
| N1 | 588 (31.39%) | 93 (38.27%) | 215 (29.37%) |  |
| N2 | 563 (30.06%) | 67 (27.57%) | 309 (42.21%) |  |
| N3 | 127 (6.78%) | 20 (8.23%) | 107 (14.62%) |  |
| Distant metastasis | 175 (9.27%) | 25 (10.29%) | 156 (21.34%) | <0.001 |
| Pathological type (N%) | | | | 0.252 |
| adenocarcinoma | 1426 (78.74%) | 181 (74.79%) | 540 (74.59%) |  |
| signet-ring cell | 357 (19.71%) | 54 (22.31%) | 171 (23.62%) |  |
| neuroendocrine | 14 (0.77%) | 4 (1.65%) | 7 (0.97%) |  |
| Other | 14 (0.77%) | 3 (1.24%) | 6 (0.83%) |  |
| Location (N%) | | | | <0.001 |
| gastric corpus | 682 (37.66%) | 59 (24.38%) | 290 (40.11%) |  |
| gastric antrum | 557 (30.76%) | 83 (34.30%) | 183 (25.31%) |  |
| whole gastric | 168 (9.28%) | 32 (13.22%) | 110 (15.21%) |  |
| esophagogastric junction cancer | 404 (22.31%) | 68 (28.10%) | 140 (19.36%) |  |
| blood type (N%) | | | | 0.67 |
| O | 731 (40.39%) | 86 (35.39%) | 299 (41.07%) |  |
| A | 527 (29.12%) | 82 (33.74%) | 215 (29.53%) |  |
| B | 420 (23.20%) | 54 (22.22%) | 164 (22.53%) |  |
| AB | 132 (7.29%) | 21 (8.64%) | 50 (6.87%) |  |
| WBC (10^9/L) | 6.58 (2.63) | 6.72 (2.11) | 6.9 (3.58) | 0.0345 |
| N (10^9/L) | 3.93 (1.98) | 4.1 (1.88) | 4.36 (3.3) | 0.0002 |
| Ly (10^9/L) | 1.92 (1.23) | 1.87 (0.67) | 1.8 (0.73) | 0.0276 |
| RBC (10^12/L) | 4.5 (9.71) | 5.5 (22.5) | 4.4 (5.11) | 0.329 |
| PLT (10^9/L) | 262.93 (95.51) | 268.45 (92.04) | 271.51 (100.4) | 0.1759 |

Chi-square test and ANOVA or Kruskal-Wallis test when nonparametric were used

Abbreviations: WBC, White blood cell count; N, Neutrophil count; Ly, Lymphocyte count; RBC, Red blood cell count; PLT, Platelet count;

| TableS3 Overall and stratified analyses of efficacy with antihypertensive and antidiabetic therapy on mortality risk | | | | | | | | | | |
| --- | --- | --- | --- | --- | --- | --- | --- | --- | --- | --- |
| Groups | Antihypertensive medications | | | | | Antidiabetic medications | | | | |
|  | Deaths/Patients | HR | 95%CI | | p | Deaths/Patients | HR | 95%CI | | p |
| Overall | 465/809 | 0.44 | 0.34 | 0.59 | <0.001 | 700/975 | 0.29 | 0.22 | 0.39 | <0.001 |
| Gender | | | | | | | | | | |
| Males | 343/603 | 0.48 | 0.35 | 0.65 | <0.001 | 490/687 | 0.27 | 0.19 | 0.39 | <0.001 |
| Females | 122/206 | 0.35 | 0.19 | 0.63 | <0.001 | 210/288 | 0.34 | 0.19 | 0.60 | <0.001 |
| Age | | | | | | | | | | |
| <50 | 145/272 | 0.13 | 0.02 | 0.97 | 0.046 | 114/152 | 0.40 | 0.13 | 1.23 | 0.109 |
| >50 | 300/498 | 0.46 | 0.35 | 0.61 | 0 | 586/823 | 0.28 | 0.21 | 0.39 | <0.001 |
| Smoking | | | | | | | | | | |
| Y | 83/148 | 0.60 | 0.27 | 1.33 | 0.208 | 130/176 | 0.43 | 0.17 | 1.11 | 0.081 |
| N | 382/661 | 0.43 | 0.32 | 0.57 | <0.001 | 570/799 | 0.28 | 0.20 | 0.38 | <0.001 |
| Drinking | | | | | | | | | | |
| Y | 27/51 | 3.18 | 0.32 | 32.14 | 0.327 | 31/49 | 0.39 | 0.04 | 3.78 | 0.415 |
| N | 438/758 | 0.43 | 0.32 | 0.57 | <0.001 | 669/926 | 0.29 | 0.21 | 0.39 | <0.001 |
| TNM stage | | | | | | | | | | |
| I/II | 43/212 | 0.70 | 0.23 | 2.17 | 0.54 | 57/174 | 0.13 | 0.03 | 0.57 | 0.007 |
| III/IV | 422/597 | 0.50 | 0.37 | 0.69 | <0.001 | 643/801 | 0.35 | 0.25 | 0.48 | <0.001 |
| T | | | | | | | | | | |
| T1/T2 | 24/144 | 0.54 | 0.13 | 2.17 | 0.385 | 24/144 | 0.60 | 0.14 | 2.66 | 0.505 |
| T3/T4 | 441/665 | 0.49 | 0.36 | 0.66 | <0.001 | 670/864 | 0.30 | 0.22 | 0.42 | <0.001 |
| N | | | | | | | | | | |
| N0/N1 | 198/472 | 0.39 | 0.25 | 0.60 | <0.001 | 268/472 | 0.21 | 0.13 | 0.36 | <0.001 |
| N2 | 209/272 | 0.57 | 0.34 | 0.94 | 0.029 | 315/375 | 0.39 | 0.22 | 0.68 | 0.001 |
| N3 | 58/65 | 0.62 | 0.05 | 7.00 | 0.697 | 117/128 | 0.42 | 0.11 | 1.63 | 0.209 |
| M | | | | | | | | | | |
| M0 | 360/697 | 0.42 | 0.31 | 0.58 | <0.001 | 522/789 | 0.27 | 0.19 | 0.37 | <0.001 |
| M1 | 105/112 | 0.95 | 0.33 | 2.72 | 0.929 | 178/186 | 0.40 | 0.15 | 1.10 | 0.076 |
| Location | | | | | | | | | | |
| gastric corpus | 170/307 | 0.22 | 0.12 | 0.43 | <0.001 | 249/348 | 0.12 | 0.05 | 0.30 | <0.001 |
| gastric antrum | 107/222 | 0.66 | 0.34 | 1.27 | 0.216 | 164/266 | 0.18 | 0.08 | 0.42 | <0.001 |
| whole gastric | 66/85 | 0.28 | 0.03 | 2.54 | 0.258 | 120/142 | 0.23 | 0.07 | 0.83 | 0.024 |
| esophagogastric junction cancer | 122/195 | 0.21 | 0.08 | 0.59 | 0.003 | 167/219 | 0.29 | 0.13 | 0.64 | 0.002 |
| Pathological type | | | | | | | | | | |
| adenocarcinoma | 352/628 | 0.35 | 0.25 | 0.49 | <0.001 | 501/720 | 0.27 | 0.19 | 0.39 | <0.001 |
| Non-adenocarcinoma | 113/181 | 0.68 | 0.32 | 1.45 | 0.322 | 199/255 | 0.13 | 0.05 | 0.37 | <0.001 |
| Tumor size | | | | | | | | | | |
| <4 cm | 58/189 | 0.98 | 0.39 | 2.45 | 0.967 | 86/186 | 0.30 | 0.12 | 0.79 | 0.014 |
| >4 cm | 407/620 | 0.43 | 0.32 | 0.59 | <0.001 | 614/789 | 0.34 | 0.25 | 0.48 | <0.001 |
| Embolus | | | | | | | | | | |
| Y | 239/335 | 0.54 | 0.33 | 0.87 | 0.011 | 389/473 | 0.42 | 0.27 | 0.67 | <0.001 |
| N | 226/474 | 0.39 | 0.26 | 0.59 | <0.001 | 311/502 | 0.15 | 0.09 | 0.27 | <0.001 |
| Differentiated | | | | | | | | | | |
| High/ Middle | 159/313 | 0.39 | 0.22 | 0.67 | 0.001 | 197/321 | 0.17 | 0.08 | 0.37 | <0.001 |
| Low | 306/496 | 0.40 | 0.27 | 0.59 | <0.001 | 503/654 | 0.25 | 0.17 | 0.37 | <0.001 |
| Blood lipid composition | | | | | | | | | | |
| Normal | 50/97 | 0.36 | 0.07 | 1.84 | 0.22 | 61/98 | 0.00 | 0.00 | . | 1 |
| Dyslipidemia | 415/712 | 0.49 | 0.36 | 0.65 | <0.001 | 639/877 | 0.29 | 0.21 | 0.39 | <0.001 |
| Fasting glucose | | | | | | | | | | |
| Non-elevated | 146/383 | 0.43 | 0.24 | 0.76 | 0.004 | 210/328 | 0.34 | 0.14 | 0.83 | 0.018 |
| Elevated | 319/426 | 0.49 | 0.34 | 0.70 | <0.001 | 490/647 | 0.19 | 0.13 | 0.27 | <0.001 |
| BMI | | | | | | | | | | |
| Normal (18-24) | 237/431 | 0.52 | 0.33 | 0.84 | 0.007 | 367/506 | 0.25 | 0.16 | 0.42 | <0.001 |
| Abnormal (<18,>24) | 228/378 | 0.43 | 0.27 | 0.66 | <0.001 | 333/469 | 0.27 | 0.17 | 0.44 | <0.001 |
| WBC | | | | | | | | | | |
| WBC<4 or NE<2 | 22/55 | #VALUE! | 0.00 | . | 1 | 15/20 | 1.00 | - | - | - |
| WBC4-10 and NE>2 | 398/688 | 0.42 | 0.31 | 0.58 | <0.001 | 599/830 | 0.28 | 0.20 | 0.39 | <0.001 |
| WBC>10 | 45/63 | 0.22 | 0.02 | 2.42 | 0.214 | 58/85 | 0.00 | 0.00 | . | 1 |
| HBG | | | | | | | | | | |
| Anemia | 199/291 | 0.34 | 0.20 | 0.57 | <0.001 | 296/378 | 0.17 | 0.08 | 0.33 | <0.001 |
| Non-anemia | 261/509 | 0.50 | 0.34 | 0.73 | <0.001 | 404/597 | 0.29 | 0.19 | 0.44 | <0.001 |
| PLT | | | | | | | | | | |
| <300 | 323/582 | 0.47 | 0.34 | 0.66 | <0.001 | 469/665 | 0.31 | 0.21 | 0.45 | <0.001 |
| >300 | 142/226 | 0.35 | 0.18 | 0.66 | 0.001 | 231/310 | 0.21 | 0.11 | 0.43 | <0.001 |
| Blood type | | | | | | | | | | |
| O | 194/333 | 0.42 | 0.25 | 0.69 | 0.001 | 284/384 | 0.25 | 0.14 | 0.43 | <0.001 |
| A | 125/239 | 0.58 | 0.30 | 1.10 | 0.098 | 203/297 | 0.21 | 0.10 | 0.44 | <0.001 |
| B | 112/175 | 0.76 | 0.37 | 1.56 | 0.455 | 167/218 | 0.27 | 0.11 | 0.67 | 0.005 |
| AB | 34/62 | 3.65 | 0.37 | 35.83 | 0.267 | 46/76 | 0.39 | 0.04 | 4.16 | 0.437 |

Abbreviations: Drinking: alcohol use history; Smoking: tobacco use history; BMI, Body mass index; Location, primary tumor location; WBC, White blood cell count; HBG, hemoglobin concentration; PLT, Platelet count; Y, yes; N, No;

| TableS4 efficacy of per medication on Cancer-specific Survival | | | | | | | | | |
| --- | --- | --- | --- | --- | --- | --- | --- | --- | --- |
| **groups** | **medication** | **HR** | **CI95%** | | **P** | **HR*** | **CI95%*** | | **P*** |
| **HT** | untreated | 1.00 |  |  |  | 1.00 |  |  |  |
|  | CCB | 0.44 | 0.33 | 0.58 | <0.01 | 0.33 | 0.22 | 0.50 | <0.01 |
|  | Other Antihypertensive | 0.86 | 0.71 | 1.04 | 0.12 | 0.71 | 0.51 | 0.99 | 0.04 |
|  | Unclear | 0.89 | 0.81 | 0.98 | 0.02 | 0.82 | 0.72 | 0.94 | 0.01 |
| **DM** | untreated | 1.00 |  |  |  | 1.00 |  |  |  |
|  | Metformin | 0.23 | 0.18 | 0.30 | <0.01 | 0.16 | 0.11 | 0.25 | <0.01 |
|  | Other Antidiabetics | 0.71 | 0.59 | 0.85 | <0.01 | 0.78 | 0.62 | 0.98 | 0.04 |
|  | Unclear | 0.76 | 0.63 | 0.93 | 0.01 | 0.87 | 0.72 | 1.04 | 0.12 |
| **HT and DM** | untreated | 1.00 |  |  |  | 1.00 |  |  |  |
|  | CCB | 0.47 | 0.34 | 0.64 | <0.01 | 0.41 | 0.24 | 0.69 | <0.01 |
|  | Other Antihypertensive | 0.75 | 0.59 | 0.95 | 0.02 | 0.55 | 0.34 | 0.89 | 0.02 |
|  | Unclear | 0.89 | 0.79 | 1.00 | 0.05 | 0.93 | 0.76 | 1.13 | 0.44 |
|  | Metformin | 0.27 | 0.19 | 0.37 | <0.01 | 0.18 | 0.09 | 0.33 | <0.01 |
|  | Other Antidiabetics | 0.73 | 0.58 | 0.92 | 0.01 | 0.76 | 0.55 | 1.05 | 0.10 |
|  | Unclear | 0.75 | 0.59 | 0.95 | 0.02 | 1.06 | 0.72 | 1.56 | 0.76 |

Abbreviations: HT, hypertension; DM, diabetes mellitus; CCB: calcium channel blockers

**FigureS1**


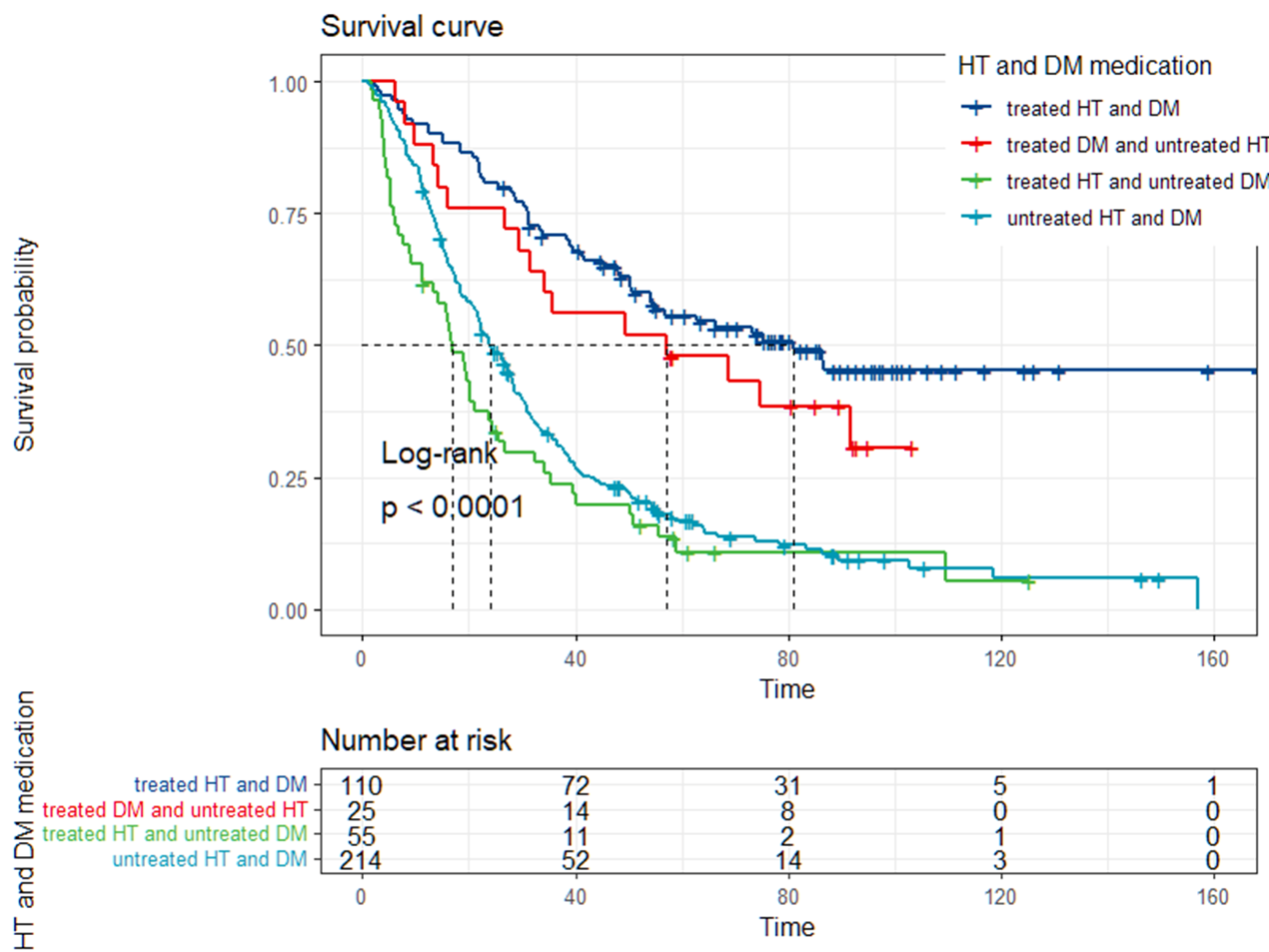


FigureS1 Combined impact of hypertension and diabetes mellitus medications on gastric cancer survival.

Abbreviations: HT, hypertension; DM, diabetes mellitus;


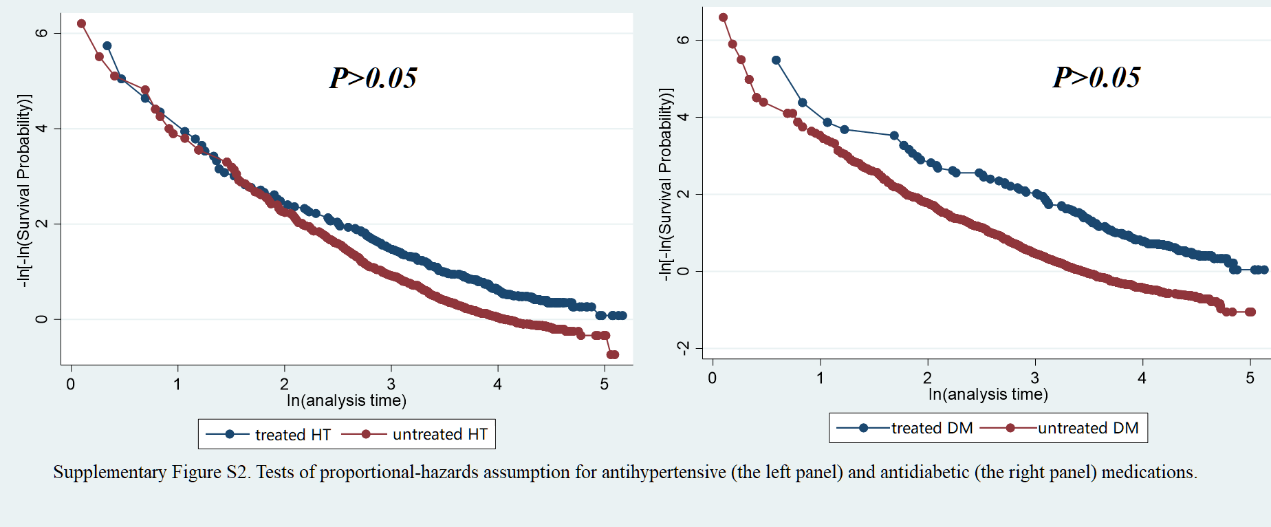


FigureS2 test of proportional-hazards assumption for antihypertensives (left panel) and antidiabetics (right panel)

Abbreviations: HT, hypertension; DM,diabetes mellitus;
